# Comparing COVID-19-related hospitalization rates among individuals with infection-induced and vaccine-induced immunity in Israel

**DOI:** 10.1038/s41467-022-29858-5

**Published:** 2022-04-22

**Authors:** Jacob G. Waxman, Maya Makov-Assif, Ben Y. Reis, Doron Netzer, Ran D. Balicer, Noa Dagan, Noam Barda

**Affiliations:** 1 Clalit Research Institute, Innovation Division, Clalit Health Services, Tel Aviv, Israel; 2 Predictive Medicine Group, Computational Health Informatics Program, Boston Children’s Hospital, Boston, MA USA; 3 Harvard Medical School, Boston, MA USA; 4 The Ivan and Francesca Berkowitz Family Living Laboratory Collaboration at Harvard Medical School and Clalit Research Institute, Boston, MA USA; 5 Community Medical Services Division, Clalit Health Services, Tel Aviv, Israel; 6 School of Public Health, Faculty of Health Sciences, Ben Gurion University of the Negev, Be’er Sheva, Israel; 7 Software and Information Systems Engineering, Ben Gurion University, Be’er Sheva, Israel; 8 Department of Biomedical Informatics, Harvard Medical School, Boston, MA USA; 9 Arc Innovation Center, Sheba Medical Center, Ramat-Gan, Israel

**Keywords:** Epidemiology, Public health, SARS-CoV-2, Vaccines

## Abstract

With the COVID-19 pandemic ongoing, accurate assessment of population immunity and the effectiveness of booster and enhancer vaccine doses is critical. We compare COVID-19-related hospitalization incidence rates in 2,412,755 individuals across four exposure levels: non-recent vaccine immunity (two BNT162b2 COVID-19 vaccine doses five or more months prior), boosted vaccine immunity (three BNT162b2 doses), infection-induced immunity (previous COVID-19 without a subsequent BNT162b2 dose), and enhanced infection-induced immunity (previous COVID-19 with a subsequent BNT162b2 dose). Rates, adjusted for potential demographic, clinical and health-seeking-behavior confounders, were assessed from July-November 2021 when the Delta variant was predominant. Compared with non-recent vaccine immunity, COVID-19-related hospitalization incidence rates were reduced by 89% (87–91%) for boosted vaccine immunity, 66% (50–77%) for infection-induced immunity and 75% (61–83%) for enhanced infection-induced immunity. We demonstrate that infection-induced immunity (enhanced or not) provides more protection against COVID-19-related hospitalization than non-recent vaccine immunity, but less protection than booster vaccination. Additionally, our results suggest that vaccinating individuals with infection-induced immunity further enhances their protection.

## Abstract (web summary)

The relative degree of immunity to SARS-CoV-2 provided by combinations of natural infection, vaccination, and booster doses is unknown. Here, the authors show that infection-induced immunity provides more protection against COVID-19-related hospitalization than non-recent vaccine immunity, but less than booster vaccination.

## Introduction

Over 2 years after the first reported case of SARS-CoV-2, the COVID-19 pandemic is still ongoing, with many countries experiencing new waves of infections and entering further lockdowns. Widespread vaccination campaigns are underway all over the world, although with extremely variable levels of population coverage^1^. Surges in healthcare utilization caused by pandemic wave peaks still pose a challenge to the capacity of healthcare systems.

Evidence of waning vaccine immunity over time has emerged: following the second vaccine, there is a significant drop in effectiveness against symptomatic infection; from a peak of ~90% in the weeks immediately following vaccination to a much lower 50–80% 6 months after vaccination^2–6^. As a result, some countries are offering booster vaccinations, amongst them Israel, in which ~4.5 million individuals have received a BNT162b2 booster dose^7^. Studies from these countries have demonstrated the benefit of booster vaccines in reducing symptomatic COVID-19 infection and providing an even greater reduction in severe outcomes^8,9^.

In contrast, the degree of protection provided by previous SARS-CoV-2 infection, also known as infection-induced immunity, remains unclear. Recent reports demonstrate what appears to be robust protection from infection-induced immunity against documented reinfection, however, both positive SARS-CoV-2 polymerase chain reaction (PCR) test and symptomatic COVID-19 as outcomes are sensitive to misclassification bias due to differential testing rates, as alluded to by the authors of these reports^10–13^. The degree of protection against outcomes that are less vulnerable to bias, such as COVID-19-related hospitalization, remains unclear. Policies regarding the management of recovered COVID-19 patients vary between countries, with some, including Israel, opting to vaccinate these individuals with a single “enhancer“ dose 3 months after diagnosis of the infection^14^. This policy is supported by the limited evidence that does exist, which demonstrates that recovered COVID-19 patients who receive a single vaccine dose have an approximately twofold reduced rate of reinfection compared to similar unvaccinated recovered individuals^11,15^. Some countries offer two vaccine doses to recovered individuals based on evidence of improved protection with a second dose^16^.

As public health officials face the challenges of continuing waves of infection, it is critical to understand how vaccine-induced immunity (with and without a booster vaccine) compares with infection-induced immunity (with and without an enhancer vaccine) in preventing COVID-19-related hospitalization. In this retrospective cohort study, we aim to shed light on this question by comparing four groups: Individuals with “non-recent vaccine immunity” (SARS-CoV-2-naive individuals with two doses of BNT162b2 and at least 5 months following the second dose and therefore eligible for a booster dose), “boosted vaccine immunity” (SARS-CoV-2-naive individuals with three doses of BNT162b2, the third dose at least 7 days previously), “infection-induced immunity” (at least 3 months following documented positive SARS-CoV-2 PCR test, and no prior or subsequent vaccination) and “enhanced infection-induced immunity” (at least 7 days from single enhancer dose of BNT162b2 administered at least 3 months following documented positive SARS-CoV-2 PCR test).

## Results

**Fig. 1:**
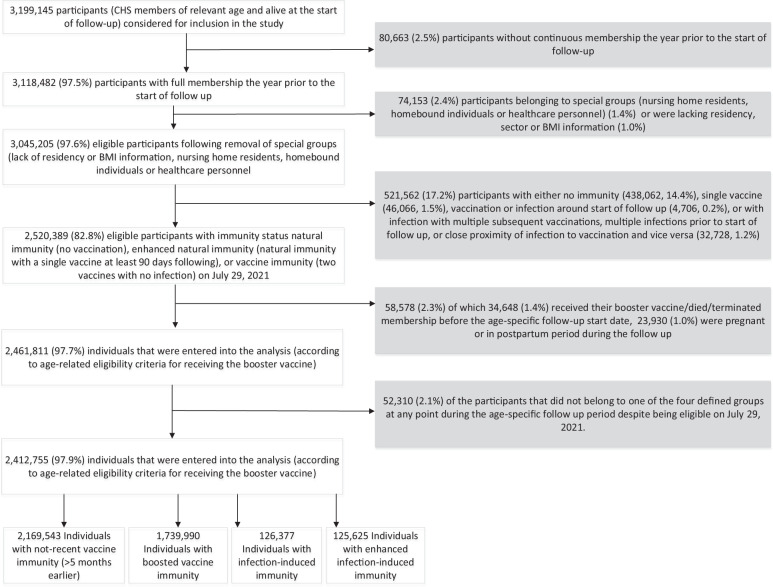
Study population flow chart. Size and percentage change of study population resulting from each inclusion and exclusion criteria.

Of a total of 3,199,145 individuals considered for the analysis, 2,412,755 (75.4%) were found eligible (Fig. 1). The median age of the study population was 47 (IQR 33–65) and 51% were female. The total time under follow-up was 235,552,274 person-days. Only 1% of cases had missing data. The number of person-days contributed varied between the groups due to differences in the number of individuals contributing to each group and the potential length of time contributed, with the most person-days contributed to boosted vaccine immunity (143,612,328), followed by non-recent vaccine immunity (72,914,787), infection-induced immunity (9,759,128), and enhanced infection-induced immunity (9,266,031). Differences of note between the exposure groups include age and disease burden (the infection-induced immunity groups are younger on average and have fewer risk factors for severe COVID-19), and population sector (the infection-induced immunity groups have a higher proportion of Ultra-Orthodox Jewish and Arab individuals). A description of the study population, stratified by exposure groups, is included in Table 1. Density plots of person-days contributed during each week of follow-up across exposure levels and exposure dates are presented in Supplementary Fig. 1A, B respectively.

**Table 1:** Baseline characteristics of study population following application of all eligibility criteria.

Characteristic^1^	Non-recent vaccine immunity, *N* = 2,169,543^a^ (72,914,787 person-days at risk)	Boosted vaccine immunity, *N* = 1,739,990^a^ (143,612,328 person-days at risk)	Infection-induced immunity, *N* = 126,3771^a^ (9,759,128 person-days at risk)	Enhanced infection-induced immunity, *N* = 125,6251^a^ (9,266,031 person-days at risk)
Age	46 (32,64)	49 (35, 67)	34 (24, 47)	38 (26, 52)
*Sex*				
Female	1,103,228 (51%)	885,688 (51%)	68,176 (54%)	68,549 (55%)
Male	1,066,315 (49%)	854,302 (49%)	58,201 (46%)	57,076 (45%)
*Sector*				
General Jewish	1,604,473 (74%)	1,396,001 (80%)	64,290 (51%)	67,958 (54%)
Ultra-orthodox	70,871 (3.3%)	53,231 (3.1%)	19,948 (16%)	13,607 (11%)
Arab	494,199 (23%)	290,758 (17%)	42,139 (33%)	44,060 (35%)
*CDC risk factors*				
0	1,066,576 (49%)	807,761 (46%)	75,151 (59%)	68,987 (55%)
1	532,955 (25%)	429,798 (25%)	31,396 (25%)	31,735 (25%)
2	262,200 (12%)	226,325 (13%)	10,473 (8.3%)	12,699 (10%)
3	155,463 (7.2%)	138,738 (8.0%)	4745 (3.8%)	6338 (5.0%)
4	86,772 (4.0%)	78,285 (4.5%)	2547 (2.0%)	3319 (2.6%)
5	41,851 (1.9%)	37,761 (2.2%)	1227 (1.0%)	1574 (1.3%)
6	23,726 (1.1%)	21,322 (1.2%)	838 (0.7%)	973 (0.8%)
*Residency type*				
Large city	822,888 (38%)	681,510 (39%)	46,877 (37%)	44,286 (35%)
Small city	778,757 (36%)	606,594 (35%)	50,071 (40%)	48,333 (38%)
Town	329,110 (15%)	241,092 (14%)	22,652 (18%)	24,427 (19%)
Village	156,215 (7.2%)	133,623 (7.7%)	5,942 (4.7%)	6779 (5.4%)
Kibbutz	82,573 (3.8%)	77,171 (4.4%)	835 (0.7%)	1800 (1.4%)
Flu vaccines in the past 5 years	1,048,397 (48%)	932,457 (54%)	33,779 (27%)	47,147 (38%)
Number of diagnoses recorded in outpatient setting (age-adjusted percentile)	0.45 (0.20, 0.73)	0.48 (0.22, 0.73)	0.62 (0.41, 0.82)	0.66 (0.45, 0.84)
Length of follow-up in days	18 (7, 48)	89 (64, 102)	94 (44, 100)	87 (45, 100)

^1^Median (IQR); *n* (%).

^a^Individuals can appear in more than one column if their exposure changed during the study.

**Fig. 2:**
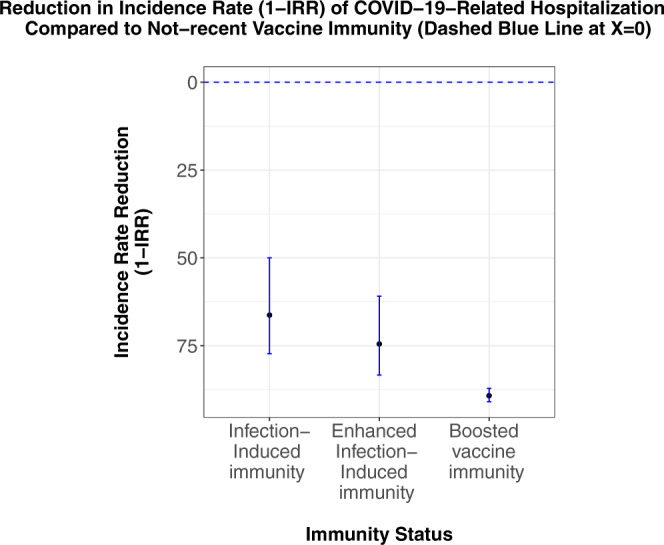
Reduction in incidence rate of COVID-19-related hospitalization compared to non-recent vaccine immunity (dashed blue line at *x* = 0). Data are presented as point estimates of the percentage reduction in incidence rate (1−IRR) and 95% confidence intervals. *N* (person-days at risk) = 143,612,328 for boosted vaccine immunity, 9,759,128 for infection-induced immunity, 9,266,031 for enhanced infection-induced immunity and 72,914,787 for non-recent vaccine immunity (reference, dashed blue line).

Compared with individuals with non-recent vaccine immunity, we estimate that the incidence rate of COVID-19-related hospitalization is reduced by 89% (87–91%) in individuals with boosted vaccine immunity, 66% (50–77%) in individuals with infection-induced immunity, and 75% (61–83%) in individuals with enhanced infection-induced immunity (Table 2 and Fig. 2).

**Table 2:** Estimated crude and adjusted incidence rate reduction of covid-19-related hospitalization for each exposure.

Immunity status	Person-days of follow-up	Events	Incidence rate	Crude estimate^a^	Adjusted estimate^a^
Non-recent vaccine immunity (≥5 months from Second Vaccine)	72,914,787	766	0.0000105	Reference	
Boosted vaccine immunity	143,612,328	213	0.0000015	86% (84–88%)	89% (87–91%)
Infection-induced immunity	9,759,128	26	0.0000027	75% (63–83%)	66% (50–77%)
Enhanced infection-induced immunity	9,266,031	22	0.0000024	77% (65–85%)	75% (61–83%)

^a^Crude and adjusted estimates of the reduction in incidence rate as compared with the reference, calculated as 1−IRR.

We performed a sensitivity analysis, in which the enhanced infection-induced immunity group included only those who received an enhancer vaccine dose less than 5 months ago (recent enhanced infection-induced immunity). The incidence rate of COVID-19-related hospitalization was reduced by 75% (52–87%) (Supplementary Table 2) compared with non-recent vaccine immunity.

## Discussion

In this retrospective cohort study of over two million individuals, we estimate that infection-induced immunity, with or without an enhancer dose, provides superior protection against COVID-19-related hospitalization compared to non-recent vaccine immunity (two vaccine doses at least five months previously) with an incidence rate reduction of 66% and 75%, respectively. Our analysis goes further to demonstrate that individuals with boosted vaccine immunity have even higher levels of protection against COVID-19 (incidence rate reduction of 89% compared with non-recent vaccine immunity). Evidence of the relative protection afforded by boosted vaccine immunity and infection-induced immunity (with or without an enhancer dose) against COVID-19-related hospitalization is sparse.

Our demonstration that infection-induced immunity provides superior protection to non-recent vaccine immunity confirms findings reported (albeit with low certainty due to the small sample size^11^ and presentation of unadjusted rates limited to individuals over 60 years old^10^) in two recent pre-print articles. We note that a small case-control study by the Centers for Disease Control and Prevention (CDC) suggested that two-dose vaccine-induced immunity is superior to infection-induced immunity^13^, however, a more recent, larger study by the CDC corroborated the findings in our study^12^. The initial study may have been affected by the inclusion of recently vaccinated individuals.

Evidence supporting the use of enhancer doses in recovered SARS-CoV-2 patients exists, but is limited^11,15^. The results of our study suggest that individuals with infection-induced immunity may benefit from an enhancer dose. The two studies to date comparing individuals with infection-induced immunity and enhanced infection-induced immunity on COVID-19-related hospitalization have very small sample sizes. In one of the studies, most individuals had received two vaccine doses following infection (as per the CDC recommendations) and only a small number had received only one enhancer dose. In that study, a 1.5-fold reduction in the odds ratio was reported for a single enhancer dose and a 2.3-fold reduction for two vaccine doses. The estimated incidence rate reduction compared with non-recent vaccine immunity in this study (75% with an enhancer dose and 66% without an enhancer dose) are consistent with the current evidence.

The case for universal booster vaccines remains a contentious issue^17^ despite a number of recent publications demonstrating a substantial risk reduction in COVID-19-related outcomes from booster vaccines^8,9^. We corroborate previous publications, showing that individuals with non-recent vaccine immunity benefit from a booster dose, with much-increased protection against COVID-19-related hospitalization (incidence rate reduction of 89%). Furthermore, the magnitude of our estimated effect size for the benefit of a booster vaccine is in line with the previous studies^8,9^.

We feel that the chosen outcome—COVID-19-related hospitalization—represents the best compromise between minimizing bias (largely circumventing the misclassification arising from differential testing rates across exposure groups common with less severe outcomes such as PCR-confirmed COVID-19 and symptomatic COVID-19) and maximizing statistical power (relative to more severe and less common outcomes such as severe COVID-19 illness and COVID-19-related death).

Our study is subject to a number of limitations. First, as in any observational study, and despite the attempts to rigorously control for potential confounders, there is still the possibility of residual confounding. Second, we are focusing on the first three months following booster vaccination—likely the period with peak immunity. It is possible that booster immunity wanes over time and further studies will be required to assess the protection from booster vaccination over the longer term. Furthermore, we compare individuals with peak immunity from a recent booster vaccination to individuals with infection-induced immunity possibly attained over a year previously or enhanced infection-induced immunity up to 8 months following the enhancer dose (Supplementary Fig. 1B). It could be argued that this is an unfair comparison and that we should instead compare recently boosted individuals to individuals who were recently infected or recently received an enhancer dose. In order to assess the validity of our study design, we performed a sensitivity analysis in which the enhanced infection-induced immunity group was limited to only include follow-up time within 5 months of the enhancer dose (recently enhanced infection-induced immunity) and repeated our analysis. The result, an identical point estimate for the risk reduction of 75% compared with non-recent vaccine immunity, with a wider confidence interval (52%–87%) reflecting the reduced sample size, supports our assertion that the results presented do not simply represent waning of the infection-induced immunity groups (Supplementary Table 2). Third, the median follow-up time in the non-recent vaccine immunity group is shorter than the other three groups (18 days compared with ~90 days), due to the rapid and high uptake of the third vaccine amongst individuals once they became eligible to receive it. This resulted in slightly different periods of follow-up for this group (majority in August 2021) compared with the booster vaccine immunity group (August–November 2021) (Supplementary Fig. 1A). While this could result in confounding by calendar time, we believe that our adjustment for the weekly local COVID-19 burden minimizes the risk of this bias. Lastly, it must be remembered that the specific exposures that we assessed in this study are vaccination with BNT162b2 and infection with non-Delta variants of SARS-CoV-2 and the specific outcome is hospitalization due to infection with the Delta variant. The results may not be generalizable to different settings and different vaccines, and specifically not when the original infection and the outcome are both from the same variant (e.g., Delta). Indeed, it is known that there is a certain amount of heterogeneity with regards to infection-induced immunity, for example, variation in the immune response to infection with different variants, which could affect the generalizability of the results^18^.

In conclusion, we demonstrate that, while infection-induced immunity (with or without an enhancer dose of BNT162b2) provides more protection against COVID-19-related hospitalization than non-recent vaccine immunity, booster vaccination provides an even greater level of relative protection. Furthermore, our results are consistent with individuals with infection-induced immunity benefiting from receipt of an enhancer dose of BNT162b2, although with a smaller relative benefit than that of the booster vaccine in the non-recent vaccine immunity group. We believe these findings can help inform members of the public and decision-makers around the world, both strengthening their conviction in the benefit and necessity of booster vaccination campaigns and enabling them to assess, more accurately, levels of COVID-19 immunity in the population.

## Methods

### Data

We analyzed observational data from Clalit Health Services (CHS), a nationwide healthcare organization that insures over 4.7 million individuals, over half of the Israeli population. The CHS-covered population is approximately representative of the general Israeli population. CHS provides all outpatient and some inpatient care to its members. CHS began to use electronic medical records over 20 years ago, with data stored centrally. All national COVID-19 data, including PCR test results, diagnoses, severity classification, and vaccinations, are collected centrally by the Israeli Ministry of Health and shared, daily, with the four national healthcare organizations, including CHS.

### Study Design and Population

This retrospective cohort study analyzed data from 30 July 2021 through November 30, 2021, coinciding with the period of the booster vaccine campaign in Israel, when the Delta variant was dominant. We compared four exposures: “non-recent vaccine immunity”, “boosted vaccine immunity”, “infection-induced immunity” and “enhanced infection-induced immunity”. The outcome of interest was COVID-19-related hospitalization. The use of a 3-month window for defining SARS-CoV-2 reinfection is in line with the CDC definition^19^.

Baseline eligibility criteria were assessed on 30 July 2021 and further eligibility criteria were assessed daily throughout the study period, with individuals only contributing time to follow-up upon meeting both baseline and additional criteria. Baseline eligibility included: age 16 years or older, and continuous membership in the healthcare organization for at least one year. In a similar manner to previous studies^20,21^, we excluded individuals belonging to populations in which confounding could not be adequately addressed—specifically healthcare workers, residents of long-term care facilities, house-bound individuals, and individuals for whom data on body-mass index, sector or residential area were missing (rare in the CHS data). In addition, we excluded pregnant women or women in the postpartum period (6 weeks following birth) from the relevant period of follow-up. Specifically for the two infection-induced immunity groups, only individuals for whom at least 3 months had passed since their SARS-CoV-2 infection on 30 July 2021, were included in order to ensure that the original infection in the infection-induced immunity exposure groups was not from the Delta variant, which came to prevalence in Israel during June 2021^22^.

Individuals meeting the above eligibility criteria were included dynamically in the study population during any day of the study period in which they met the exposure definitions (for example, once 5 months had elapsed from their second dose). Exposure was determined in a time-varying manner, such that individuals were able to contribute time to multiple exposure groups. For example, an individual could contribute time as “non-recent vaccine immunity” until the date the individual received a booster vaccine, and as “boosted vaccine immunity” from 7 days following the booster vaccine. Likewise with infection-induced immunity and enhanced infection-induced immunity.

In order to ensure that all individuals included in the analysis could receive all exposure levels, individuals were considered “under analysis” only once their age group was eligible to receive the booster dose. The booster vaccine campaign in Israel was rolled out in a stepwise manner, with booster vaccines being offered to increasingly younger age-groups as follows: Individuals aged 60 and older from 30 July 2021; individuals aged 50 to 59 from 12 August 2021; individuals aged 40 to 49 from 19 August 2021; individuals aged 30–39 from 24 August 2021; and individuals aged 16–29 from 30 August 2021. (Hence, for example, individuals aged 50–59, were only eligible to start follow-up from 12 August 2021.)

We adjusted for a wide variety of potential confounders, determined by domain expertise to be associated with both the exposure (type of immunity) and the outcome (COVID-19-related hospitalization). These variables included age (as a continuous variable), sex (male or female), week number of follow-up (as a continuous variable), type of residency (a six-level categorization including large city, small city, town, village and kibbutz [a type of communal village specific to Israel]), COVID-19 burden by location of residency (defined as the percentage of total tests in the previous week that were positive in a specific residency code), population sector (general Jewish, ultra-orthodox Jewish or Arab), socioeconomic status (a 20-point scale treated as a continuous variable), the number of preexisting chronic conditions (those considered to be risk factors for severe COVID-19 by the Centers for Disease Control and Prevention as of 20 December 2020, divided into seven categories; 0, 1, 2, 3, 4, 5, or ≥6), and, as indicators of health-seeking behavior, number of diagnoses documented in the outpatient setting in the previous year (percentile for age group in 10 year bins) and receipt of at least one flu vaccine in the 5 years prior to the follow-up (as a binary variable). A complete definition of the study variables, including exposures, the outcome, and covariates, is included in Supplementary Table 1.

The end of follow-up for each person was defined as the earliest of the following outcomes: end of the study period, death, termination of healthcare organization membership, or occurrence of the outcome (COVID-19-related hospitalization).

### Statistical analysis

The effect estimate of interest was the reduction in incidence rate from the baseline; defined as 1−Incidence Rate Ratio (IRR). The IRR was estimated using multivariable Poisson regression, with ‘non-recent vaccine immunity’ used as the baseline level of the exposure. Continuous variables (age, week of follow-up, COVID-19 burden by location of residency, and socioeconomic status) were modeled as restricted cubic splines with 2, 3, 3, and 3 degrees of freedom, respectively.

The unit of time in the analysis was a single person-day. To allow for time-varying covariates, including a time-varying exposure (e.g., individuals receiving the booster dose during the study period), each person was represented in the data by multiple rows, each with its own covariate values, with a variable representing the number of days “at-risk” in each row used as the “offset” in the model. That means that each individual could contribute time to more than one exposure group. Each row represented a maximal follow-up of 7 person-days.

We opted to perform a complete case analysis given that missing data in the variables used are rare in the CHS dataset.

### Sensitivity analysis

In addition, we performed a sensitivity analysis in which an identical analysis to that described above was performed, but follow-up was limited to a maximum of 5 months from the date of the enhancer vaccine (recent vaccination) in the enhanced infection-induced immunity group, in an attempt to eliminate the effect of waning immunity.

### Ethics

CHS institutional review board approved this study and it was deemed exempt from the requirement for informed consent (Reference ID 0052-20-COM2).

### Reporting summary

Further information on research design is available in the Nature Research Reporting Summary linked to this article.

## Supplementary information

Supplementary Information

Reporting Summary

## Data Availability

Due to national and organizational data privacy regulations, individual-level data such as those used for this study cannot be shared openly.

## Code availability

Data collection was performed in Microsoft SQL Server Management Studio v.13.0.16100.1. The modeling in this paper used R v.4.1.0 and the tidyverse v.1.3.1 R packages, all of which are freely available. The code is available at: https://github.com/clalitresearch/native_vs_vaccine_immunity.
