# Supplementary material for: Comparing COVID-19-related hospitalization rates among individuals with infection-induced and vaccine-induced immunity in Israel
Source: Nat Commun. 2022 Apr 22;13:2202. doi: 10.1038/s41467-022-29858-5 (PMC9033865; doi:10.1038/s41467-022-29858-5)
Supplement: Supplementary file 1 — Supplementary Information [file 41467_2022_29858_MOESM1_ESM.pdf]

Comparing COVID-19-Related Hospitalization Rates Among Individuals with Infection-induced and Vaccine-induced Immunity in Israel

Jacob G. Waxman*[1], Maya Makov-Assif*[1], Ben Y. Reis[2,3,4], Doron Netzer[5], Ran D. Balicer[1,4,6], Noa Dagan[+1,4,7,8], Noam Barda[+1,4,7,8]

* These authors contributed equally
+ These authors jointly supervised this work

[1] Clalit Research Institute, Innovation Division, Clalit Health Services, Tel Aviv, Israel
[2] Predictive Medicine Group, Computational Health Informatics Program, Boston Children's Hospital, Boston, MA, USA
[3] Harvard Medical School, Boston, MA, USA
[4] The Ivan and Francesca Berkowitz Family Living Laboratory Collaboration at Harvard Medical School and Clalit Research Institute, Boston, MA, USA
[5] Community Medical Services Division, Clalit Health Services, Arlozorov 101, Tel Aviv, Israel
[6] School of Public Health, Faculty of Health Sciences, Ben Gurion University of the Negev, Be'er Sheva, Israel
[7] Software and Information Systems Engineering, Ben Gurion University, Be'er Sheva, Israel
[8] Department of Biomedical Informatics, Harvard Medical School, Boston, MA, USA

Corresponding Author:
Jacob G. Waxman
Clalit Research Institute, Innovation Division, Clalit Health Services, Tel Aviv, Israel
JacobWa@clalit.org.il

Supplementary Information – Table of Contents

Supplementary Table 1: Definitions of Variables used for the Exposure, Eligibility and Adjustment 3

Supplementary Table 2: A sensitivity analysis limited enhanced infection-induced immunity to follow-up that falls within 5 months of the date of the enhancer dose 10

Supplementary Figure 1A: Distribution of person-days contributed during each week of follow-up during the study period (August-November 2021) stratified by exposure level 11

Supplementary Figure 1B: Distribution of exposure dates (vaccination or infection) of all individuals included in the analysis 12

Supplementary Table 1 – Definitions of Variables used for the Exposure, Eligibility and Adjustment

Names, potential values, definitions and time periods for all variables used in the study to define exposure, eligibility and perform adjustment. Variables were defined using internal CHS registries, ICD-9 codes, and ATC codes. PCR: Polymerase Chain Reaction; CHS: Clalit Health Services; ICD: International Classification of Disease; ATC: Anatomic therapeutic chemical; COPD: Chronic Obstructive Pulmonary Disease.

Variable	Values	Definitions[1]	Timing[2]
**Exposure**			
Non-recent vaccine immunity	0/1	Two doses of BNT162b2 mRNA COVID-19 Vaccine at least 5 months previously	Administration of the second dose no later than May 29, 2021
Boosted vaccine immunity	0/1	Three doses of BNT162b2 mRNA COVID-19 Vaccine at least 7 days previously	Administration of the third dose after the age-related follow-up start dates: July 30, 2021 for over 60s, August 12, 2021 for over 50s, August 19, 2021 for over 40s, August 24, 2021 for over 30s, August 30, 2021 for over 16s
Infection-induced immunity	0/1	PCR-confirmed SARS-CoV-2 infection, with no previous COVID-19 vaccination	The date of specimen collection that was found to be positive in a PCR test, not after April 30,2021
Enhanced infection-induced immunity	0/1	PCR-confirmed SARS-CoV-2 infection, with a single dose of BNT162b2 at least 3 months following infection	The date of specimen collection that was found to be positive in a PCR test, not after April 30,2021
**Outcome**			
COVID-19 related hospitalization	0/1	A hospitalization that was reported to the Israeli MOH as a hospitalization of a SARS-CoV-2 infected individual.	The start date of the hospitalization
**Eligibility & Adjustment**			
Age	Integer	Age in complete years	Current
Sex	Male/ Female	As defined in CHS files	Current
Long-term care facility resident	0/1	Is patient a long-term care facility resident per CHS files	Current
Confined to Home	0/1	Is patient confined to his home per CHS files	Current
Health-care worker	0/1	Is patient a health-care worker per CHS files	Current

Continuous Membership in the Health Organization	0/1	Was patient a member of CHS for a full year before the index date	Last Year
Place of Residence	List of places	Residency code in which the patient resides per CHS files	Current
Socioeconomic Status	Integer between 1 and 20	Patient socioeconomic status, based on home address	Current
Population Sector	General Jewish / Arab / Ultraorthodox Jewish	As defined in CHS files	Current
Pregnancy up to 6 weeks post-partum	0/1	As defined in CHS files	Current
Influenza vaccinations	0/1	Did patient receive an influenza vaccination in the last 5 years	Last 5 Years
Number of diagnoses recorded in past year (age-adjusted percentile)	Integer	Number of diagnoses recorded in the outpatient setting in the past year as an age-adjusted percentile	Last year
Cancer	0/1	ICD9 Code 174*<br>ICD9 Code 175*<br>ICD9 Code 233.0<br>ICD9 Code V10.3<br>ICD9 Proc Code 85.4*<br>ICD9 Code 153*<br>ICD9 Code 154*<br>ICD9 Code V10.5*<br>ICD9 Code V10.6*<br>ICD9 Code 185<br>ICD9 Code V10.46<br>ICD9 Code 162*<br>ICD9 Code V10.1*<br>ICD9 Code 188*<br>ICD9 Code V10.51<br>ICD9 Code 183*<br>ICD9 Code V10.43<br>ICD9 Code 179<br>ICD9 Code 182*<br>ICD9 Code V10.42<br>ICD9 Code 157*<br>ICD9 Code 191*<br>ICD9 Code 192*<br>ICD9 Code V10.85<br>ICD9 Code 151*<br>ICD9 Code V10.04<br>ICD9 Code 172*<br>ICD9 Code V10.82<br>ICD9 Code 201*<br>ICD9 Code 200*<br>ICD9 Code 202.4*<br>ICD9 Code 204*	Last 5 years

		ICD9 Code 205*<br>ICD9 Code 206*<br>ICD9 Code 207.1*<br>ICD9 Code 208.1*<br>ICD9 Code 189*<br>ICD9 Code V10.52<br>ICD9 Code 160*<br>ICD9 Code 161*<br>ICD9 Code 164.0<br>ICD9 Code 195.0<br>ICD9 Code V10.21<br>ICD9 Code V10.22<br>ICD9 Code 180*<br>ICD9 Code V10.41<br>ICD9 Code 140*<br>ICD9 Code 141*<br>ICD9 Code 142*<br>ICD9 Code 143*<br>ICD9 Code 144*<br>ICD9 Code 145*<br>ICD9 Code 150*<br>ICD9 Code V10.03<br>ICD9 Code 155*<br>ICD9 Code 156*<br>ICD9 Code V10.07<br>ICD9 Code 170*<br>ICD9 Code V10.81<br>ICD9 Code 193<br>ICD9 Code V10.87<br>ICD9 Code 171*<br>ICD9 Code 176*<br>ICD9 Code 184*<br>ICD9 Code 186*<br>ICD9 Code 187*<br>ICD9 Code V10.4*<br>ICD9 Code 203*<br>ICD9 Code 273.3<br>ICD9 Code 152*<br>ICD9 Code 158*<br>ICD9 Code 159*<br>ICD9 Code 163*<br>ICD9 Code 164*<br>ICD9 Code 165*<br>ICD9 Code 181<br>ICD9 Code 190*<br>ICD9 Code 192.8<br>ICD9 Code 196*<br>ICD9 Code 197*<br>ICD9 Code 198*<br>ICD9 Code 199*	
Chronic Kidney Disease	0/1	ICD Proc Code 39.95<br>ICD Proc Code 54.98<br>ICD9 Code 996.81<br>ICD9 Code V42.0<br>ICD Proc Code 55.6*	Ever

		ICD9 Code 403._1<br>ICD9 Code 404._2<br>ICD9 Code 404._3<br>ICD9 Code 585*<br>ICD9 Code 586<br>ICD9 Code 250.4*<br>ICD9 Code 274.1*<br>ICD9 Code 440.1<br>ICD9 Code 581*<br>ICD9 Code 582*<br>ICD9 Code 583*<br>ICD9 Code 587<br>ICD9 Code 588*<br>ICD9 Code 589*	
Chronic Obstructive Pulmonary Disease	0/1	ICD9 Code 491*<br>ICD9 Code 492*<br>ICD9 Code 496	Ever
Heart Conditions	0/1	ICD9 Code 410*<br>ICD9 Code 411*<br>ICD9 Code 412<br>ICD9 Code 413*<br>ICD9 Code 414*<br>ICD9 Code 429.2, 429.7*<br>ICD9 Code V45.81, V45.82<br>ICD9 Proc Code 36.0*<br>ICD9 Proc Code 36.1*<br>ICD9 Code 428*<br>ICD9 Code 398.91<br>ICD9 Code 402._1<br>ICD9 Code 404._1, ICD9 Code 404._3<br>ICD9 Code 416.9<br>ICD9 Code 514<br>ICD9 Code 425*<br>ICD9 Code 416*	Ever
Solid Organ Transplant Recipient	0/1	ICD9 Code 996.81<br>ICD9 Code V42.0<br>ICD Proc Code 55.6*<br>ICD9 Code V42.7<br>ICD Proc Code 50.5*<br>ICD9 Code V42.1<br>ICD9 Code V43.2<br>ICD Proc Code 37.5<br>ICD9 Code V42.83<br>ICD Proc Code 52.8*<br>ICD9 Code V42.6<br>ICD Proc Code 33.5*<br>ICD Proc Code 33.6	Ever
Obesity	0/1	Body Mass Index (BMI) 30-40	Latest measurement in last 5 years not taken during pregnancy
Severe Obesity	0/1	Body Mass Index (BMI) 40+	Latest measurement in last 5 years not taken during pregnancy
Pregnancy	0/1	Internal Clalit Registry	Current

Sickle Cell Disease	0/1	ICD9 Code 282.6*	Ever
Smoking	0/1	Internal Clalit Registry	Last recorded value
Type 2 Diabetes Mellitus	0/1	HbA1C > 6.5<br>ATC Codes A10[A,B]<br>ICD9 Code 250*<br>ICD9 Code 357.2<br>ICD9 Code 362.0*<br><br>**And not:**<br>ICD9 Code 250._1, 250._3	For diagnosis codes, Ever<br>For drugs, 4 or more dispensed in last 12 months
Asthma	0/1	ICD9 Code 493*	Ever
Cerebrovascular Disease	0/1	ICD9 Code 362.34<br>ICD9 Code 430<br>ICD9 Code 431<br>ICD9 Code 432*<br>ICD9 Code 433*<br>ICD9 Code 434*<br>ICD9 Code 435*<br>ICD9 Code 436*<br>ICD9 Code 438*	Ever
Other Respiratory Disease	0/1	ICD9 Code 277.0*<br>ICD9 Code 494*<br>ICD9 Code 515	Ever
Hypertension	0/1	ICD9 Code 401*<br>ICD9 Code 402*<br>ICD9 Code 403*<br>ICD9 Code 404*<br>ICD9 Code 405*	Ever
Immunocompromised State	0/1	**Any of:**<br>ICD9 Code 042*<br>ICD9 Code 043*<br>ICD9 Code 044*<br>ICD9 Code 795.71<br>ICD9 Code V08<br>ICD9 Code V42.8*<br>ICD9 Proc Code 41.0*<br><br>**Or at least 2 of:**<br>ATC4 Code H02AB<br>ATC4 Code H02BX<br>ATC4 Code M01BA<br><br>**Or at least 2 of:**<br>ATC2 Code L04	For diagnosis codes, Ever<br>For drugs, 4 or more dispensed in last 12 months
Neurologic Conditions	0/1	ICD9 Code 290.*<br>ICD9 Code 294*<br>ICD9 Code 310.1<br>ICD9 Code 331*<br>ATC Codes N06DA02, N06DA03<br>ICD9 Code 358*<br>ICD9 Code 332.[0,1]<br>ICD9 Code 345*<br>ICD9 Code 340	For diagnosis codes, Ever<br>For drugs, 4 or more dispensed in last 12 months

		ATC Codes L03AB07, L03AB08, L04AA07<br>ICD9 Code 343*<br>ICD9 Code 333.4<br>ICD9 Code 334*<br>ICD9 Code 356*<br>ICD9 Code 138<br>ICD9 Code 335*<br>ICD9 Code 730.7*<br>ICD9 V12.02<br>ICD9 Code 228.02<br>ICD9 Code 307.23<br>ICD9 Code 330.9<br>ICD9 Code 331.3*<br>ICD9 Code 331.4<br>ICD9 Code 333*<br>ICD9 Code 334*<br>ICD9 Code 336*<br>ICD9 Code 337<br>ICD9 Code 335.1*<br>ICD9 Code 359.0<br>ICD9 Code 359.21<br>ICD9 Code 357.0<br>ICD9 Code 237.7*<br>ICD9 Code 742.8[1,2]	
Liver Disease	0/1	ICD9 Code 070.22<br>ICD9 Code 070.23<br>ICD9 Code 070.32<br>ICD9 Code 070.33<br>ICD9 Code 070.44<br>ICD9 Code 070.54<br>ICD9 Code V02.61<br>ICD9 Code V02.62<br>ICD9 Code 571*<br>ICD9 Code 275.1<br>ICD9 Code 277.4<br>ICD9 Code 452<br>ICD9 Code 453.0<br>ICD9 Code 571.8<br>ICD9 Code 571.9<br>ICD9 Code 572*	Ever
Overweight	0/1	Body Mass Index (BMI) 25-30	Latest measurement in last 5 years not taken during pregnancy
Thalassemia	0/1	ICD9 Code 282.4*	Ever
Type 1 Diabetes Mellitus	0/1	ICD9 Code 250._1, 250._3	Ever
Count of pre-existing conditions considered by the CDC as risk criteria	Integer	Summation of 1 point for each of the following:<br>• Cancer<br>• CKD<br>• Heart Disease<br>• Sickle Cell Disease<br>• Asthma<br>• Cerebrovascular Disease	Based on the variables above

		• Hypertension<br>• Neurological Disease<br>• Liver Disease<br>• Thalassemia<br>• COPD or other respiratory disease<br>• Type 1 or 2 Diabetes Mellitus<br>• Solid organ transplant recipient or immunodeficiency<br>• Obesity or severe obesity	

[1]Additional confirmation of the diagnostic codes was done by checking the matching of the free text within the diagnosis description field.

[2] Covariates were extracted at the beginning of the calendar month in which the index date occurred.

Supplementary Table 2: A sensitivity analysis limited enhanced infection-induced immunity to follow-up that falls within 5 months of the date of the enhancer dose

Immunity status	Person-Days of Follow up	Events	Incidence Rate	Crude Estimate*	Adjusted Estimate*
Non-recent Vaccine Immunity (>=5 months from Second Vaccine)	72,914,787	766	0.0000105	Reference	
Boosted Vaccine Immunity	143,612,328	213	0.0000015	86% (84%-88%)	89% (87%-91%)
Infection-induced immunity	9,759,128	26	0.0000027	75% (63%-83%)	66% (50%-77%)
Recently Enhanced Infection-induced immunity (within 5 months)	4,930,915	9	0.0000018	83% (66%-91%)	75% (52%-87%)

* Crude and adjusted estimates of the reduction in incidence rate as compared with the reference, calculated as 1 – IRR

Supplementary Figure 1A: Distribution of person-days contributed during each week of follow-up during the study period (July-November 2021) stratified by exposure level

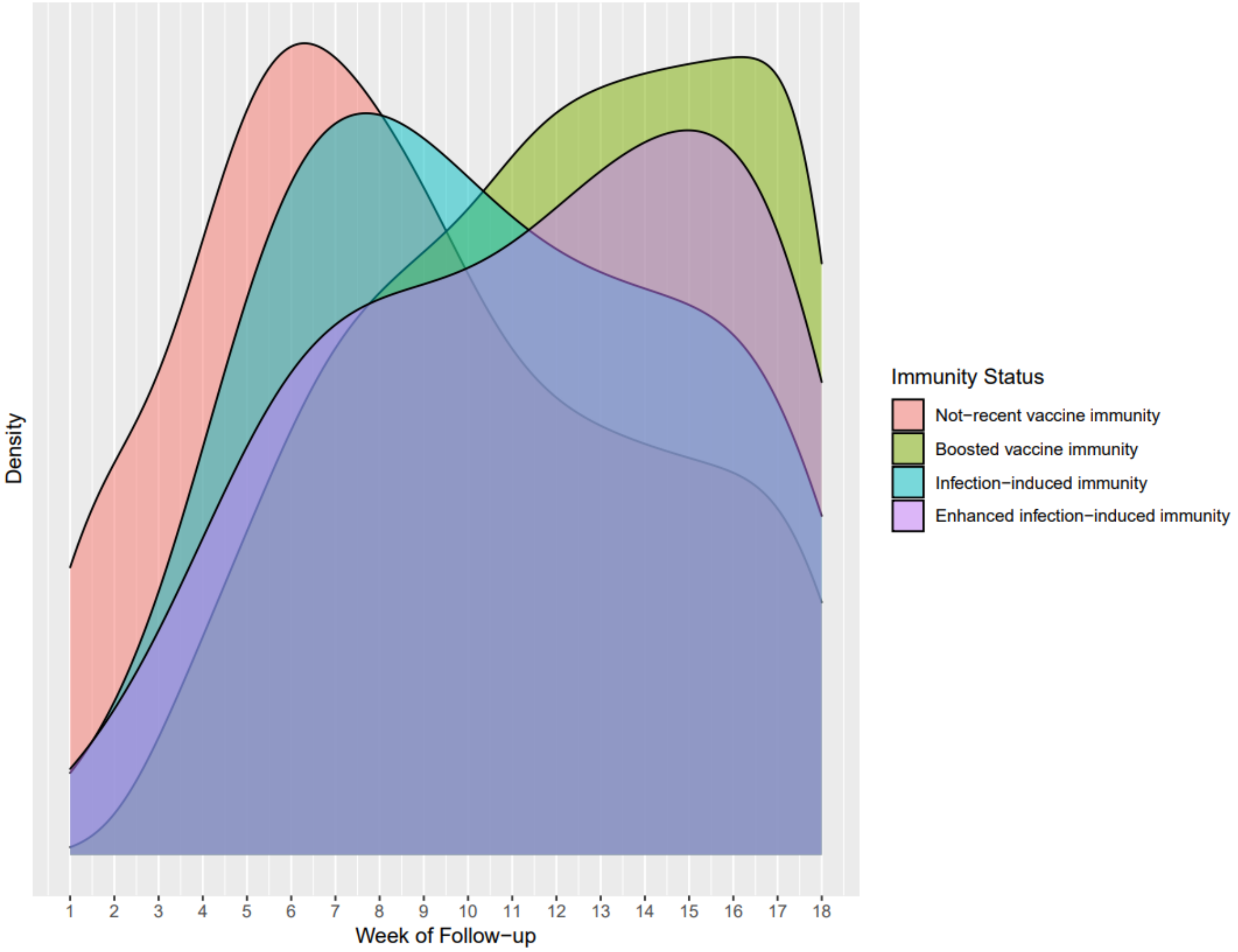

The study period, presented according to week of follow-up on the x-axis, includes dates from July 30, 2021 until November 30, 2021.

Supplementary Figure 1B: Distribution of exposure dates (vaccination or infection) of all individuals included in the analysis

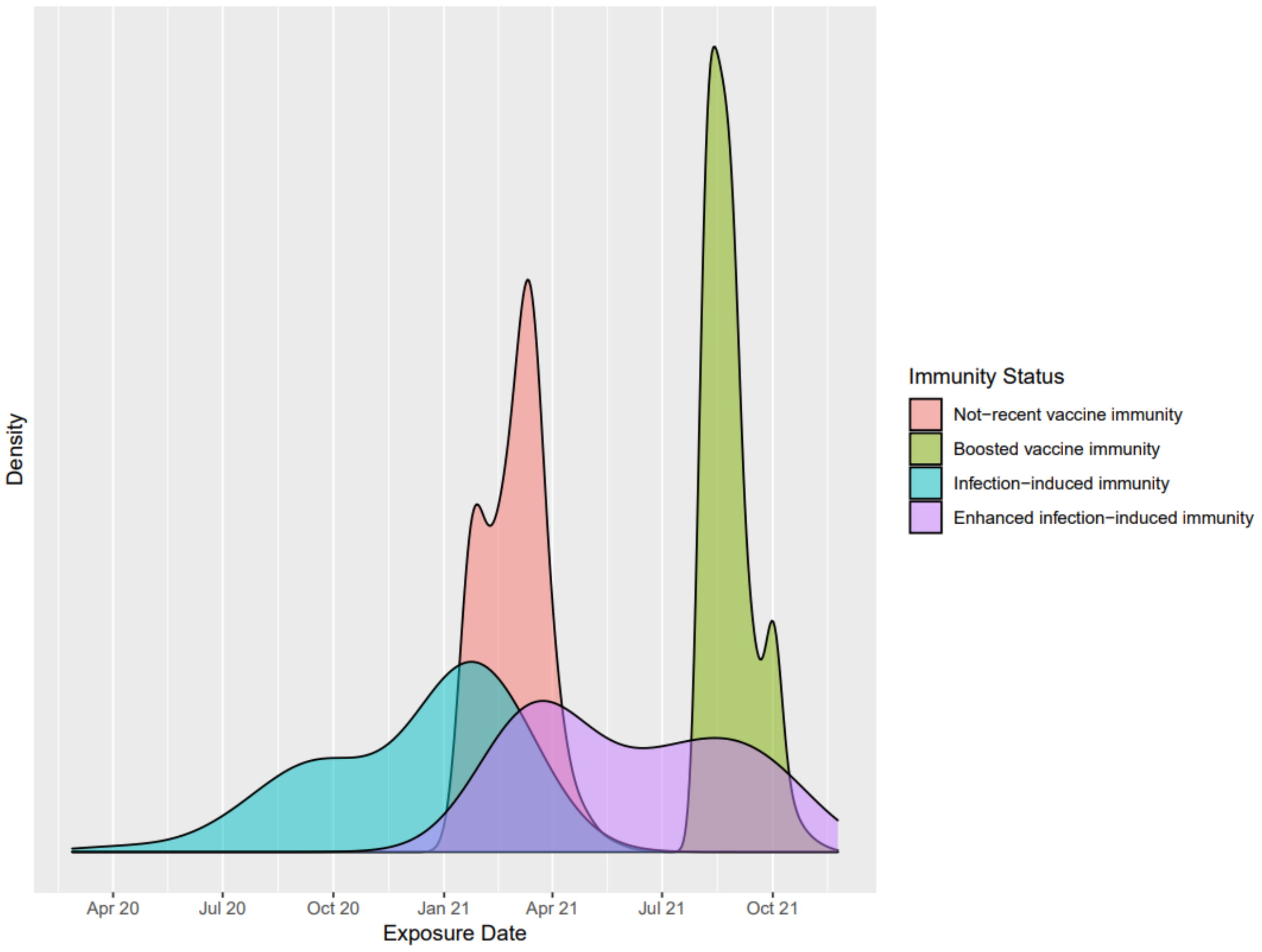

Exposure dates are defined as the date of the second vaccine dose in the non-recent vaccine immunity group, the date of the third vaccine dose in the boosted vaccine immunity group, the date of SARS-CoV-2 infection for individuals in the infection-induced immunity group and the date of the enhancer vaccine in the enhanced infection-induced immunity group.
