# Supplementary material for: Comparing COVID-19-related hospitalization rates among individuals with infection-induced and vaccine-induced immunity in Israel
Source: Nat Commun. 2022 Apr 22;13:2202. doi: 10.1038/s41467-022-29858-5 (PMC9033865; doi:10.1038/s41467-022-29858-5)
Supplement: Supplementary file 2 — Reporting Summary [file 41467_2022_29858_MOESM2_ESM.pdf]

nature portfolio

Corresponding author(s): Jacob G. Waxman

Last updated by author(s): Mar 10, 2022

Reporting Summary

Nature Portfolio wishes to improve the reproducibility of the work that we publish. This form provides structure for consistency and transparency in reporting. For further information on Nature Portfolio policies, see our Editorial Policies and the Editorial Policy Checklist.

Statistics

For all statistical analyses, confirm that the following items are present in the figure legend, table legend, main text, or Methods section.

n/a	Confirmed	
☐	☒	The exact sample size (*n*) for each experimental group/condition, given as a discrete number and unit of measurement
☐	☒	A statement on whether measurements were taken from distinct samples or whether the same sample was measured repeatedly
☐	☒	The statistical test(s) used AND whether they are one- or two-sided<br>*Only common tests should be described solely by name; describe more complex techniques in the Methods section.*
☐	☒	A description of all covariates tested
☒	☐	A description of any assumptions or corrections, such as tests of normality and adjustment for multiple comparisons
☐	☒	A full description of the statistical parameters including central tendency (e.g. means) or other basic estimates (e.g. regression coefficient) AND variation (e.g. standard deviation) or associated estimates of uncertainty (e.g. confidence intervals)
☒	☐	For null hypothesis testing, the test statistic (e.g. *F*, *t*, *r*) with confidence intervals, effect sizes, degrees of freedom and *P* value noted<br>*Give P values as exact values whenever suitable.*
☒	☐	For Bayesian analysis, information on the choice of priors and Markov chain Monte Carlo settings
☒	☐	For hierarchical and complex designs, identification of the appropriate level for tests and full reporting of outcomes
☒	☐	Estimates of effect sizes (e.g. Cohen's *d*, Pearson's *r*), indicating how they were calculated

Our web collection on statistics for biologists contains articles on many of the points above.

Software and code

Policy information about availability of computer code

Data collection	SQL Server V.13.0.16100.1
Data analysis	R v.4.1.0 and the tidyverse v.1.3.1 R package

For manuscripts utilizing custom algorithms or software that are central to the research but not yet described in published literature, software must be made available to editors and reviewers. We strongly encourage code deposition in a community repository (e.g. GitHub). See the Nature Portfolio guidelines for submitting code & software for further information.

Data

Policy information about availability of data

All manuscripts must include a data availability statement. This statement should provide the following information, where applicable:

- Accession codes, unique identifiers, or web links for publicly available datasets
- A description of any restrictions on data availability
- For clinical datasets or third party data, please ensure that the statement adheres to our policy

Due to national and organizational data privacy regulations, individual-level data such as those used for this study cannot be shared openly.

Field-specific reporting

Please select the one below that is the best fit for your research. If you are not sure, read the appropriate sections before making your selection.

☐ Life sciences ☒ Behavioural & social sciences ☐ Ecological, evolutionary & environmental sciences

For a reference copy of the document with all sections, see nature.com/documents/nr-reporting-summary-flat.pdf

Behavioural & social sciences study design

All studies must disclose on these points even when the disclosure is negative.

Study description	This is a retrospective cohort analysis, based on existing medical records. Methods are strictly quantitative.
Research sample	The population of this study is the patient population of a large integrated healthcare provider operating in Israel. Individuals 16 years or older, with continuous membership in the healthcare organization for at least a year and meeting both the exposure definitions during the relevant (age-group specific) study period as defined in the methods section. Median age of the study population was 47 (IQR 33-65) and 51% were female. Differences of note between the exposure groups include: Age and disease burden (the infection-induced immunity groups are younger on average and have fewer CDC risk factors), and population sector (the infection-induced immunity groups have a higher proportion of Ultra-Orthodox Jewish and Arab individuals). We present the full set of characteristics of the population stratifiied by exposure group in Table 1.
Sampling strategy	Due to the retrospective nature of this study all qualified members were included and no sample-size calculation was done.
Data collection	All data used in this study is based on existing electronic medical records. No dedicated data collection was performed. Data was extracted using SQL according to the inclusion and exclusion criteria defined. Given the retrospective nature of the study, no blinding was performed.
Timing	The study period is from July 30, 2021 through November 30, 2021. Background variables were extracted from the period prior to inclusion in the study.
Data exclusions	As detailed in Figure S1, the following individuals were excluded: individuals without continuous membership in the healthcare organization for at least a year, healthcare workers, residents of long-term care facilities, house-bound individuals, individuals for whom data on body-mass index, sector or residential area were missing, pregnant women or women in the 6 weeks postpartum and individuals that did not meet one of the four defined exposures at any point during the relevant (age-group specific) study period.
Non-participation	None of the 2,412,755 participants who met the eligibility criteria were not included in the analysis.
Randomization	This is an observational study, no randomization was performed.

Reporting for specific materials, systems and methods

We require information from authors about some types of materials, experimental systems and methods used in many studies. Here, indicate whether each material, system or method listed is relevant to your study. If you are not sure if a list item applies to your research, read the appropriate section before selecting a response.

Materials & experimental systems

n/a	Involved in the study	
☒	☐	Antibodies
☒	☐	Eukaryotic cell lines
☒	☐	Palaeontology and archaeology
☒	☐	Animals and other organisms
☐	☒	Human research participants
☒	☐	Clinical data
☒	☐	Dual use research of concern

Methods

n/a	Involved in the study	
☒	☐	ChIP-seq
☒	☐	Flow cytometry
☒	☐	MRI-based neuroimaging

Human research participants

Policy information about studies involving human research participants

Population characteristics	See above
Recruitment	Participants were not recruited, but rather selected from a retrospective Clalit medical database. All eligible participants were considered. As with any observational study, there exists the potential for residual bias, even after adjustment for potential confounders. Furthermore, as mentioned in the discussion, there is potential for confounding by calendar time (the rapid

	uptake of the booster dose resulted in the majority of follow-up in the non-recent vaccine immunity group being in August compared with the follow up for the booster vaccine group being August-November) although we believe this is mitigated by our adjustment for weekly local COVID-19 burden.
Ethics oversight	This study was approved by the CHS institutional review board and it was deemed exempt from the requirement for informed consent

Note that full information on the approval of the study protocol must also be provided in the manuscript.
